# Catechin attenuates TNF-α induced inflammatory response via AMPK-SIRT1 pathway in 3T3-L1 adipocytes

**DOI:** 10.1371/journal.pone.0217090

**Published:** 2019-05-17

**Authors:** An-Wei Cheng, Xin Tan, Jin-Yue Sun, Chun-Mei Gu, Chao Liu, Xu Guo

**Affiliations:** 1 Institute of Agro-food Science and Technology, Shandong Academy of Agricultural Sciences, Jinan, China; 2 Key Laboratory of Novel Food Resources Processing, Ministry of Agriculture, Jinan, China; 3 Key Laboratory of Agro-Products Processing Technology of Shandong Province, Jinan, China; 4 College of Food Science and Engineering, Jilin Agricultural University, Changchun, China; National Institutes of Health, UNITED STATES

## Abstract

Chronic inflammation is a fundamental symptom of many diseases. Catechin possesses anti-oxidant and anti-inflammatory properties. However, the mechanism of catechin to prevent inflammation in 3T3-L1 adipocytes caused by TNF-α remains unknown. Therefore, the effects of catechin on the gene expression of cytokines and the activation of cell signals in TNF-α induced 3T3-L1 adipocytes were investigated. The effects of catechin on adipogenesis and cell viability were detected by Oil Red O staining and CCK-8 assay, respectively. The genes expression of cytokines was determined by real-time RT-PCR. The expression of NF-κB, AMPK, FOXO3a and SIRT1 on translation level was determined by western blotting analysis. The results demonstrated that catechin significantly enhanced adipogenesis and cell viability. catechin inhibited the gene expression of pro-inflammatory cytokines including IL-1α, IL-1β, IL-6, IL-12p35, and inflammatory enzymes including iNOS and COX-2, but enhanced the gene expression of anti-inflammatory cytokines including IL-4 and IL-10. Catechin also inhibited the activation of NF-κB, AMPK, FOXO3a and SIRT1, but increased the phosphorylation level of the above factors. All these results indicated that as a potential therapeutic strategy catechin has the ability of attenuating inflammatory response triggered by TNF-α through signaling cascades involved in inflammation and cytokines.

## Introduction

Inflammation is a vital survival mechanism in human but it would be dangerous when it loses balance in metabolism and survives, and may slowly develop into a chronic state. Several metabolic disorders, such as obesity, diabetes and atherosclerosis, stir up immune defense mechanisms and stimulate chronic inflammation which in turn aggravate the symptoms of the diseases [1–3]. Especially in recent years obesity has become a worldwide health problem. Obesity can lead to inflammation, and its induced insulin resistance is the key to the occurrence of metabolic syndrome. Some studies have shown that adipose tissue/cells are not only the place for energy storage, but also secrete a variety of adipokines and inflammatory factors [4], and there is a certain relationship between energy metabolism and immune regulation at the cellular level. The formation of inflammatory mediators is a highly energy-consuming process, and the energy metabolism state of cells is closely related to the occurrence and development of inflammation. The inflammatory effects of tumor necrosis factor (TNF)-α are triggered by the activation of the inflammatory signaling networks, including nuclear factor (NF)-κB, AMP-activated protein kinase (AMPK), forkhead box O3a (FOXO3a), sirtuin1 (SIRT1) pathways in key metabolic tissues as well as adipocytes [5–9], which is responsible for the increasing of pro-inflammatory cytokines, such as interleukin (IL)-1, IL-6, IL-12, and inflammatory enzymes, such as inducible nitric oxide synthase (iNOS) and cyclooxygenase-2 (COX-2), but for the decreasing of anti-inflammatory cytokines, such as IL-4 and IL-10 [6,7,10–13].

AMPK consists of of a catalytic subunit (α) and two regulatory subunits (β, γ), which is activated by the phosphorylation of the threonine residue (Thr172) [14]. It is a key regulator to maintain the stability of energy metabolism at the cellular level [15]. AMPK has a large number of downstream substrates which are usually metabolic enzymes and protein. SIRT1, a NAD^+^-dependent protein deacetylase, plays an important role in the regulation of physiological and pathological processes such as apoptosis/aging, metabolism, differentiation and inflammation through deacetylation of intracellular signaling factors [16, 17]. AMPK and SIRT1 have synergistic effects on maintaining evolutionary stability, and have similar roles in cellular metabolism and survival [18]. Canto *et al*. [19] demonstrated that AMPK can activate SIRT1 deacetylase by increasing cellular NAD^+^ levels. In contrast, SIRT1 can also activate AMPK by stimulating the activity of LKB1 [20]. Through such an effective feedback loop to replenish the energy shortage during cell survival.

The commonly studied dietary polyphenols have been reported to possess antioxidant and anti-inflammatory properties. Polyphenols are the main functional compounds of tea and there are a class of flavonoids known as catechins (the most abundant component) which comprise of (–)-epigallocatechin-3-gallate, (–)-epigallocatechin, (–)-epicatechin, and (–)-epicatechin-3-gallate [21]. The previous reports about the anti-inflammatory activity and mechanism of catechin were mainly using lipopolysaccharide (LPS)-induced macrophages [22–24], rarely were found using TNF-α induced 3T3-L1 adipocytes. Mouse 3T3-L1 preadipocytes are commonly recognized as a cell model to study differentiation and metabolism of adipocyte. Therefore, we took 3T3-L1 preadipocytes as the model to study the relationship between energy metabolism and inflammatory response at the cellular level. TNF-α is a major player mediating the activation of signaling cascades in adipocytes that are central to inflammation and insulin resistance [25]. It has been widely known that the target of AMPK/SIRT1 plays a key role in maintaining metabolic homeostasis [18], which is a ubiquitous regulator of mitochondrial activity, but little has been known about its anti-inflammtory regulatory role in inflammatory adipocytes. Therefore, in the present study the role of catechin in the prevention of TNF-α mediated inflammation resistance in 3T3-L1 adipocytes were investigated.

## Materials and methods

### Materials

Fetal bovine serum (FBS), Dulbecco's Modified Eagle's Medium (DMEM) and penicillin-streptomycin solution were purchased from Gibco Laboratory (Carlsbad, CA, USA). Trizol reagent was purchased from Invitrogen Life Technologies (Carlsbad, CA, USA). Cell Counting Kit-8 (CCK-8) was purchased from Dojindo Laboratorise (Kumamoto, Japan). catechin (> 97%), 3-isobutyl-1-methylxanthine (IBMX), insulin, dexamethasone (DEX), TNF-α and Oil Red O were ordered from Sigma-Aldrich Company (St. Louis, MO, USA). HyPure^TM^ Molecular Biology Grade Water, phosphate buffer solution (PBS) were purchased from Hyclone (Waltham, MA, USA). The reagents for electrophoretic mobility shift assays were from SunShine Biotechnology (Hangzhou, China). RevertAid First Strand cDNA Synthesis Kit was from Thermo Scientific (Waltham, MA, USA). FastStart Universal SYBR Green Master (Rox) was from Roche (Basel, Switzerland). Antibodies for NF-κB-p65, p-NF-κB-p65 (Ser276) were from Abcam Inc. (Cambridge, MA); SIRT1, p-SIRT1 (Ser27), FOXO3a, p-FOXO3a (Ser253) and glyceraldehyde-3-phosphate dehydrogenase (GAPDH) were from ABclonal Biotechnology (Woburn, MA, USA); AMPK and p-AMPK(Thr172) were from Cell Signaling Technology (Danvers, MA, USA). Cell culture dishes and multi-well plates were supplied by Nunc (Denmark).

### Cell culture and differentiation

3T3-L1 preadipocytes obtained from China Center of Type Culture Collection (CCTCC, Wuhan, China) were cultured in DMEM supplemented with 10% FBS, 100 g/mL streptomycin, 100 U/mL penicillin, 44 mM NaHCO_3_ and 1 mM sodium pyruvate at 37°C in a 5% CO_2_ humidification atmosphere (Thermo Scientific BBD6220, Germany). To induce differentiation, the cultured 3T3-L1 preadipocytes were kept until confluence was reached (0 day), and the culture medium was then replaced with a fresh induction medium containing 0.5 mM IBMX, 10 μg/mL insulin and 1 μM DEX in DMEM with 10% FBS for 2 days. The cells was then cultivated in a differentiation DMEM medium containing 10 μg/mL insulin and 10% FBS for four times (2 days for each time) until the cells were harvested.

The harvested cells were cultured in a complete medium containing 10 ng/mL TNF-α for 24 h (except the control group), then the media were removed and adherent cells were washed with PBS for twice. The obtained adipocytes were incubated in complete DMEM containing catechin (0, 10, 25, 50, 100 μg/mL) up to 48 h. Finally the adipocytes were collected for following experiments.

### Oil Red O staining

Intracellular lipid accumulation was measured using Oil Red O staining. The mature 3T3-L1 adipocytes were washed twice with PBS, fixed with 10% formalin (pH 7.4) for 15 min, and washed with distilled water for 10 s. The washed adipocytes were stained with Oil Red O solution for 30 min, and then washed with distilled water for 60 s. The fat droplets in 3T3-L1 adipocytes were observed by phase contrast microscopy (CKX41, Olympus, Japan). After removing the staining solution, 200 μL isopropanol was added into the dye retained in the cells, and the value was determined by a microplate reader at absorbance of 510 nm. The value of OD_510_ may indirectly represents the content of intracellular triglyceride.

### CCK-8 assay

The cell viability was measured by using CCK-8 kits according to the manufacturer’s instruction. Absorbance of the samples was measured at 450 nm by a spectrophotometer (UV-1750, Shimadzu, Japan).

### RNA isolation and real-time PCR

Total RNA was isolated from the control and treated cells using Trizol reagent. The quantity of total RNA was determined by using a spectrophotometer according to the absorbance at A260/A280 nm. cDNA was generated by using a High Capacity RNA-to-cDNA Kit. Real-time PCR analysis was performed on a StepOne Real-time PCR system (Applied Biosystems) in triplicates by using FastStart Universal SYBR Green Master (Rox) (Roche) and cDNA as template. The specific upstream and downstream primer sequences were shown in Table 1. Cycling was initiated at 95°C for 2 min, followed by 40 cycles of 95°C for 10 s, 60°C for 40 s. The relative expression of each gene was calculated using the comparative threshold cycle method normalized to GAPDH.

**Table 1:** Primers used for RT-PCR.

Genes	Primer sequences	Expected product size (bp)
IL-1α	F-5'-TGAGTCGGCAAAGAAATCAA-3' R-5'-AGAGAGATGGTCAATGGCAGA-3'	108
IL-1β	F-5'-TGGGCTGGACTGTTTCTAATG-3' R-5'-GGTTTCTTGTGACCCTGAGC-3'	134
IL-6	F-5'-GAGGATACCACTCCCAACAGACC-3' R-5'-AAGTGCATCATCGTTGTTCATACA-3’	141
IL-12p35	F-5'-TTGATGATGACCCTGTGCCT-3' R-5'-GATTCTGAAGTGCTGCGTTGA-3'	91
IL-4	F-5'-AAGGGATACAGGGGCTCACT-3' R-5'-CAGGTCTATTCGGTGGACAAA-3'	121
IL-10	F-5'-TGGACAACATACTGCTAACCGAC-3' R-5'-ATGCTCCTTGATTTCTGGGC-3'	143
iNOS	F-5'-GAAAAGTCCAGCCGCACCAC-3' R-5'-GGACAATCCACAACTCGCTCC-3'	149
COX-2	F-5'-CTGCCAATAGAACTTCCAATCC-3' R-5'-CGGTTTGATGTTACTGTTGCTT-3'	134
GAPDH	F-5'-AGGAGCGAGACCCCACTAACA-3' R-5'-AGGGGGGCTAAGCAGTTGGT-3'	247

### Western blotting analysis

Protein extracts were prepared by lysing cells in RIPA lysis buffer, and centrifuged at 14,000 rpm for 15 min at 4°C. Protein concentration of the resultant supernatant was measured by using BCA Assay Reagent Kit (Thermo Fisher Scientific Inc., Rockford, IL, USA). Proteins in cell lysates were separated by sodium dodecyl sulfate-polyacrylamide gel electrophoresis (SDS-PAGE) on 12% gels, transferred onto a polyvinyliden fluoride membrane for 1.5 h at 250 mV, and then blocked with 5% skim milk in TBS containing 1% Tween 20 (TBST) for 1 h at room temperature. The transferred membranes were washed 3 times with TBST for 10 min, then incubated with the primary antibodies at a dilution of 1:1000 in PBS containing 2% BSA overnight at 4°C. After the incubation, the membranes were washed 3 times for 10 min each time and incubated for 1 h at room temperature in TBST, After three times of washes with TBST the membranes were finally exposed to ECL plus western blot analysis detection reagents.

### Statistical analysis

Results of three replica were expressed as mean ± standard deviation (SD). A one-way analysis of variance (ANOVA) was performed to calculate significant differences in treatment means, and multiple comparisons of means were performed by the post-hoc test (SPSS 19.0 software). A probability value of *p* < 0.05 was considered significant.

## Results

### Effects of catechin on adipogenesis and cell viability in 3T3-L1 adipocytes

**Fig 1:**
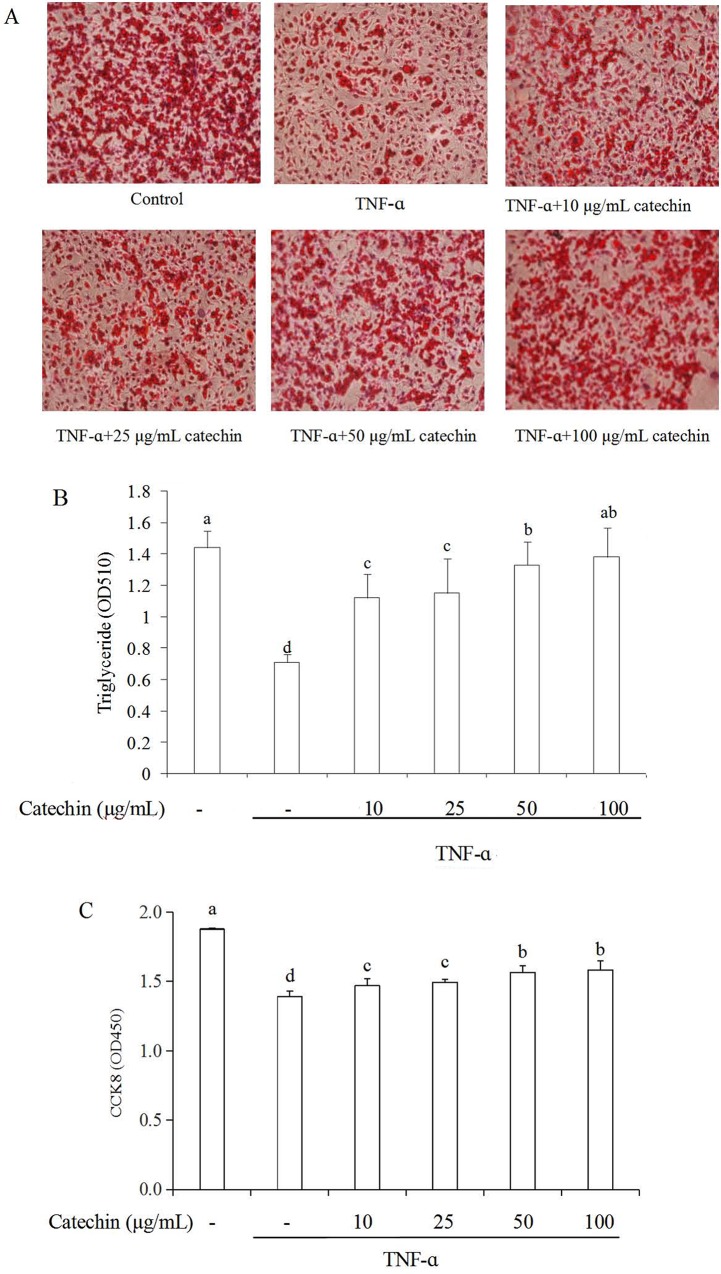
Effects of catechin on adipogenesis and cell viability in TNF-α (10 ng/mL) induced 3T3-L1 adipocytes. (A) Lipid droplets in 3T3-L1 adipocytes treated with various concentrations of catechin were stained by Oil Red O; (B) The value of OD_510_ represents the content of intracellular triglyceride; (C) Cell viability of adipocytes treated with various concentrations of catechin was assayed by CCK8. Data bars with the unlike letters represent the significance differences (*p* < 0.05), with the same letters represent no significance difference (*p* > 0.05).

The effect of catechin on cell adipogenesis was measured by Oil Red O staining as shown in Fig 1A. The density of lipid droplets in adipocytes under the condition of without the stimulation of TNF-α was high, however the density significantly decreased when adipocytes was stimulated by TNF-α, which indicated that TNF-α could induce the loss of intracellular lipids. The quantity of lipid droplets was significantly increased when the TNF-α stimulated 3T3-L1 adipocytes treated with catechin, and they are in a dose-dependent manner. In the TNF-α induced adipocytes treated with 100 μg/mL of catechin, the density of lipid droplets reached the same level as the control group. These results indicated that catechin can impair the formation of lipid droplets, and enhance intracellular lipids accumulation during differentiation. The value of OD_510_ indirectly reflects the content of intracellular triglyceride (Fig 1B). Similarly, the effects of catechin were significant at the increasing of triglyceride accumulation.

The effect of catechin on cell viability was measured by CCK-8 assay as shown in Fig 1C. The OD_450_ value of the TNF-α stimulated 3T3-L1 adipocyteds was obviously lower than that of un-stimulated adipocytes. When catechin were added in the TNF-α induced adipocytes, the OD_450_ values increased in a dose-dependent manner. These results indicated that catechin could enhance the cell viability of the TNF-α stimulated adipocytes. It was speculated that catechin was non-cytotoxic in the range of concentrations (10–100 μg/mL), therefore this concentration range of catechin was used in the subsequent experiments.

### Catechin prevent TNF-α induced activation of pro-inflammatory cytokines in 3T3-L1 adipocytes

**Fig 2:**
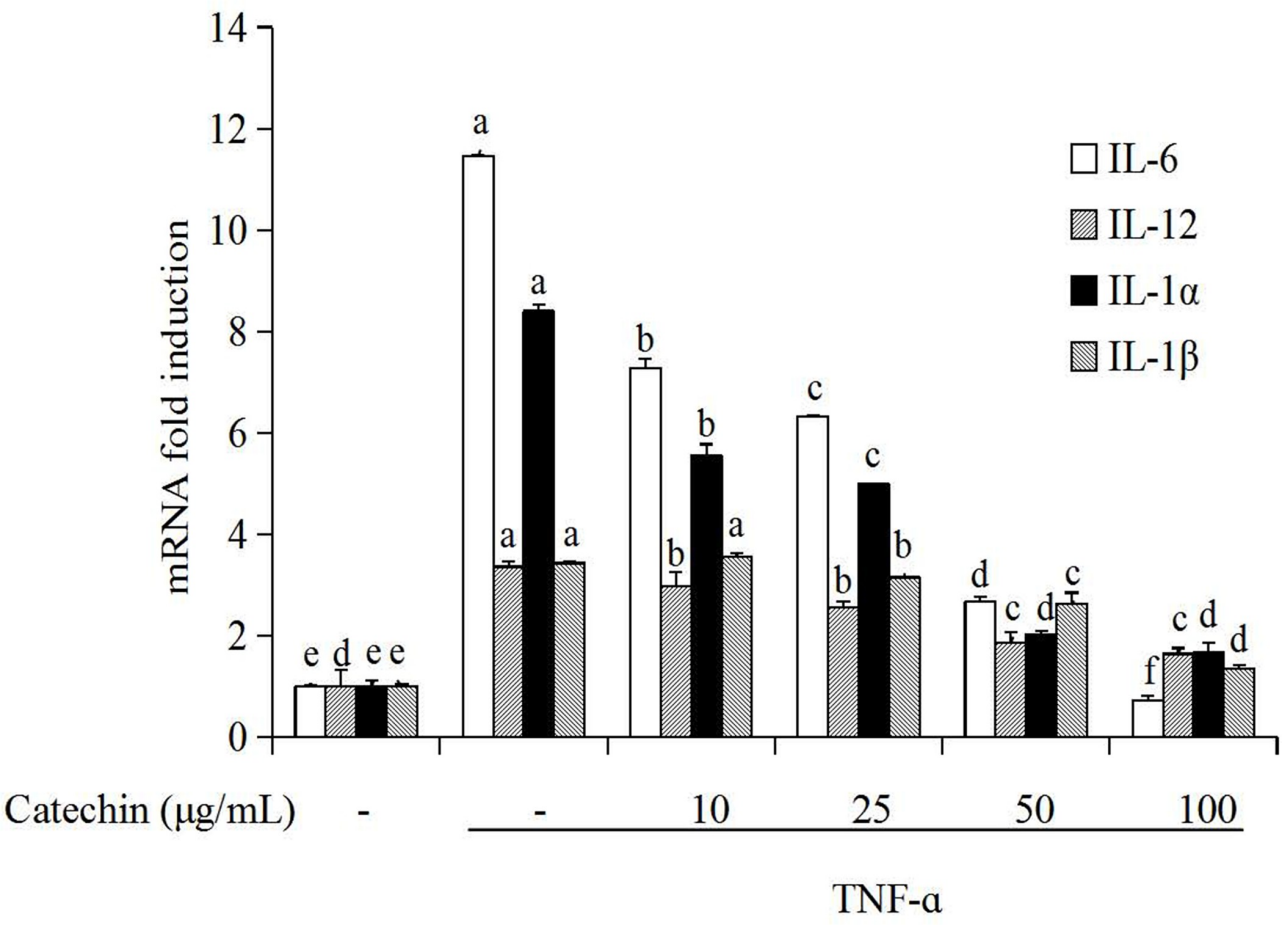
The inhibition of catechin on the expression of genes related with pro-inflammatory cytokines induced by TNF-α (10 ng/mL) induced in 3T3-L1 adipocytes. Data bars with the unlike letters represent the significance differences (*p* < 0.05), with the same letters represent no significance difference (*p* > 0.05).

Different concentrations (10, 25, 50, 100 μg/mL) of catechin were added to the TNF-α induced 3T3-L1 adipocytes and cultured for 48 h. The effects of catechin on gene expression of pro-inflammatory cytokines including IL-6, IL-12p35, IL-1α, IL-1β were shown in Fig 2. The results demonstrated that the gene expression levels of IL-6, IL-12p35, IL-1α, IL-1β were the lowest (fold induction 1) in the groups without TNF-α stimulation (control group, CK). But, the gene expression of the above aspro-inflammatory cytokines was sharply increased in adipocytes after TNF-α (10 ng/mL) stimulation, and the mRNA levels of IL-6, IL-1α, IL-1β, IL-12p35 after induction reached to 11.46, 8.40, 3.43 and 3.36 folds, respectively of the control. When catechin was added to the TNF-α induced 3T3-L1 adipocytes, the gene expression of pro-inflammatory cytokines on the transcription level significantly decreased in a dose-dependent manner. The inhibition rate was over 80% at the group with 100 μg/mL catechin. Similar tendency in the effect of catechin on gene expression of the above pro-inflammatory cytokines was observed.

### Catechin improve TNF-α induced activation of anti-inflammatory cytokines in 3T3-L1 adipocytes

**Fig 3:**
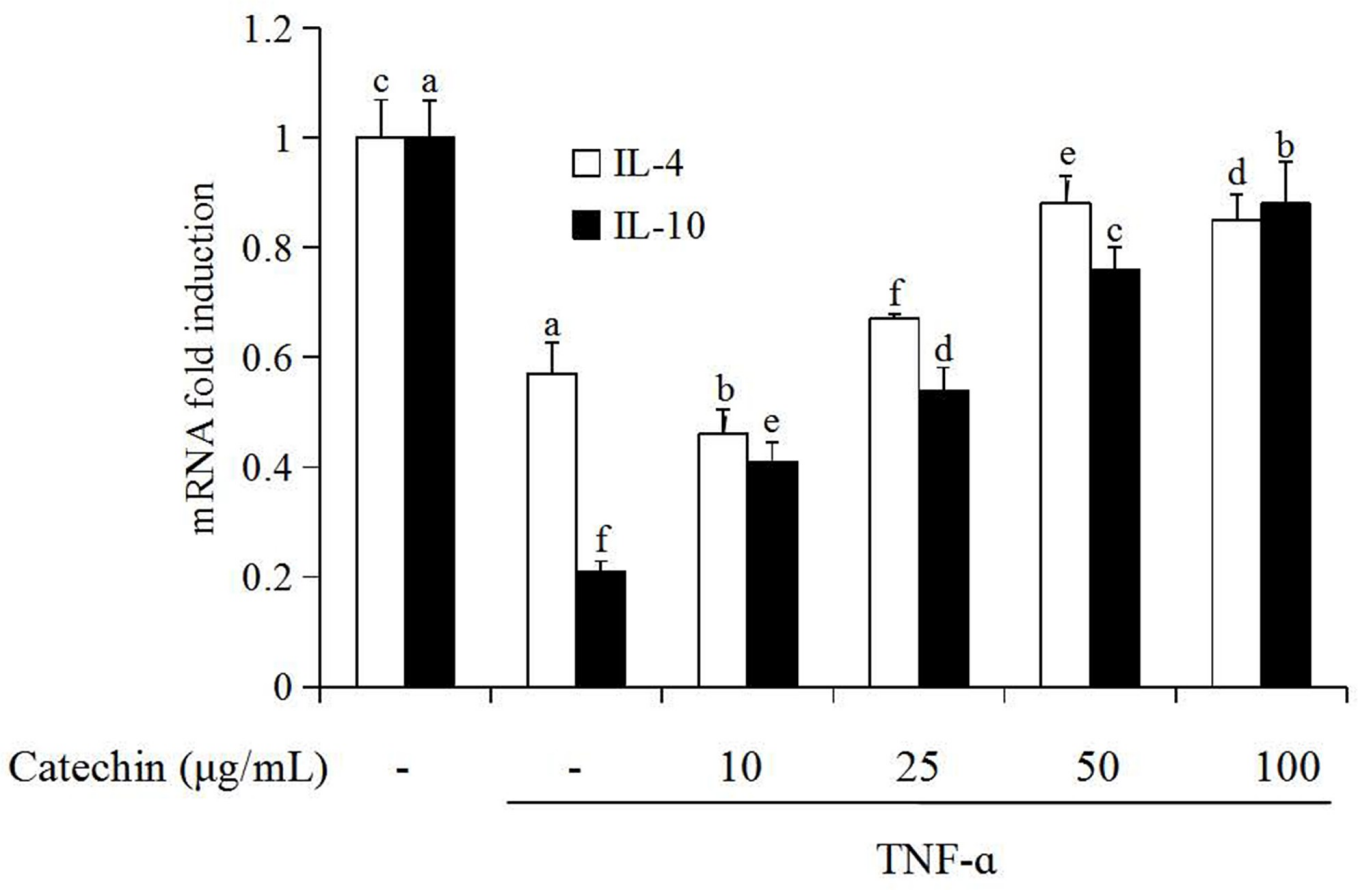
The enhancement of catechin on the expression of genes related with anti-inflammatory cytokines induced by TNF-α (10 ng/mL) in 3T3-L1 adipocytes. Data bars with the unlike letters represent the significance differences (*p* < 0.05), with the same letters represent no significance difference (*p* > 0.05).

The effect of catechin (10–100 μg/mL) on the gene expression of anti-inflammatory cytokines including IL-4 and IL-10 in 3T3-L1 adipocytes were presented in Fig 3. The expression of IL-4 and IL-10 on transcription level was the highest (fold induction 1) in adipocytes without the stimulation of TNF-α (control group, CK). When 3T3-L1 adipocytes were induced by TNF-α, the expression of IL-4 and IL-10 on transcription level decreased significantly, the fold induction of f IL-4 and IL-10 was 0.57 and 0.21 folds, respectively. When catechin was added to the media, the gene expression of IL-4 and IL-10 on the transcription level in TNF-α induced adipocytes was significantly increased (*P* < 0.05) in a dose-dependent manner. For the treatment with 100 μg/mL catechin, the transcription level of IL-4 and IL-10 reached to 2 and 4.4 folds, respectively.

### Catechin prevent TNF-α induced activation of inflammatory enzymes in 3T3-L1 adipocytes

**Fig 4:**
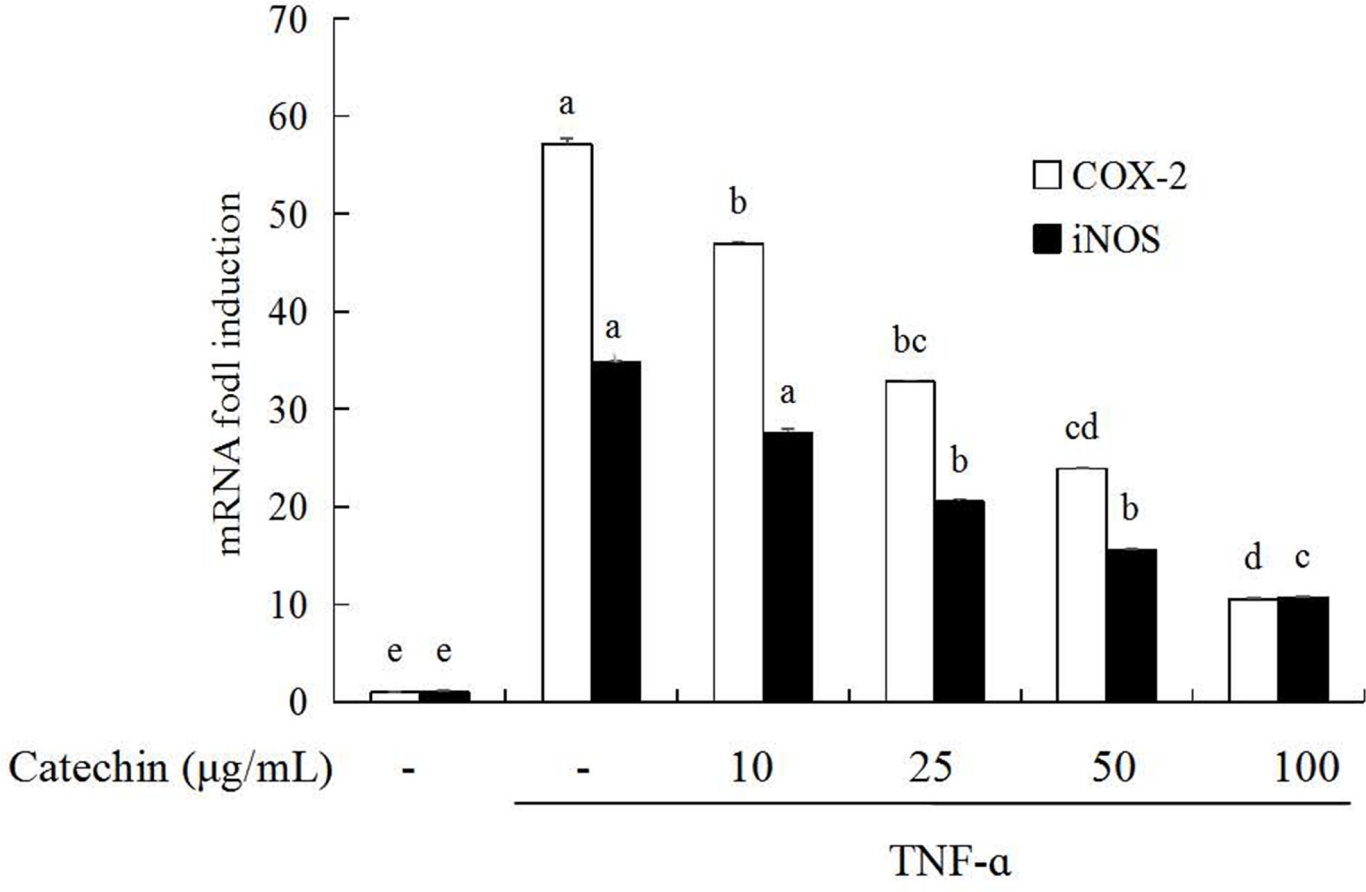
The inhibition of catechin on the expression of genes related with inflammatory enzymes induced by TNF-α (10 ng/mL) in 3T3-L1 adipocytes. Data bars with the unlike letters represent the significance differences (*p* < 0.05), with the same letters represent no significance difference (*p* > 0.05).

Inhibitory effects of catechin on gene expression of inflammatory enzymes iNOS and COX-2 in 3T3-L1 adipocytes were presented in Fig 4. When 3T3-L1 adipocytes were stimulated by TNF-α, an obvious increase of the gene expression of iNOS (fold induction 34.79) and COX-2 (fold induction 57.17) was detected. When catechin was added to the media, the iNOS and COX-2 mRNA expression decreased in a concentration-dependent manner in the range of 10 to 100 μg/mL, and the inhibitory rate of 100 μg/mL catechin on iNOS and COX-2 mRNA was over 60% and 80%, respectively.

### AMPK-SIRT1 pathway is associated with catechin in 3T3-L1 adipocytes

**Fig 5:**
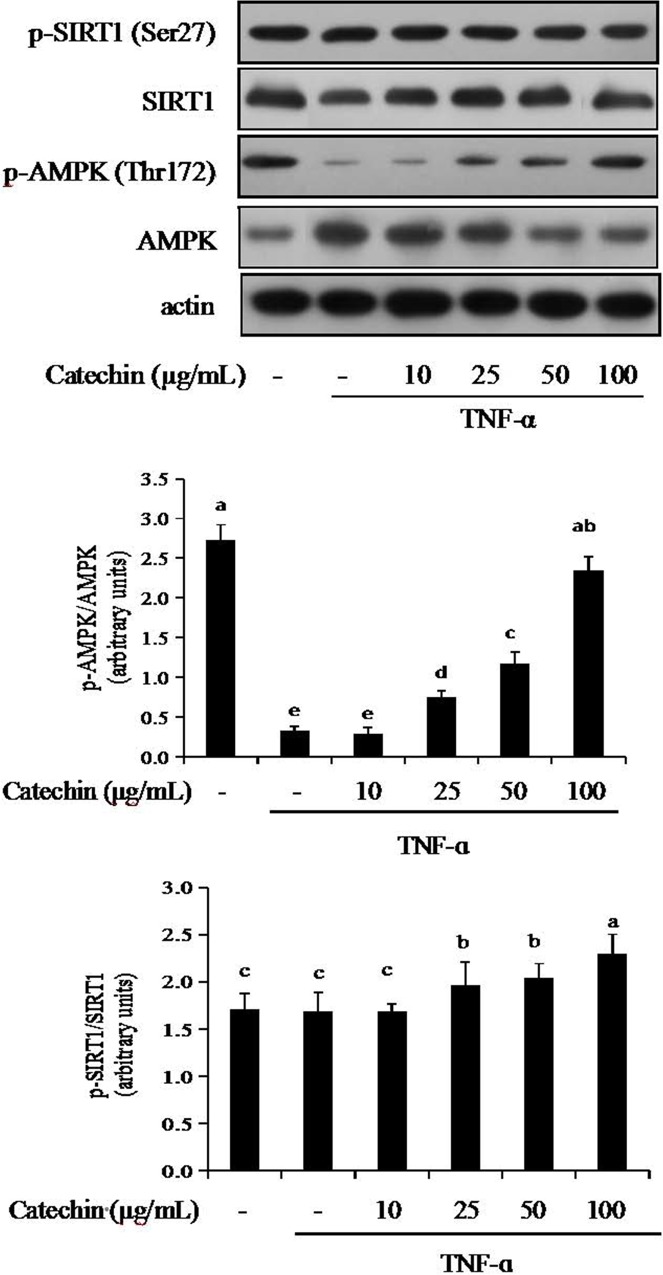
The effects of catechin on the activation of phosphorylation of AMPK and SIRT1 inhibited by TNF-α (10 ng/mL) in 3T3-L1 adipocytes. Data bars with the unlike letters represent the significance differences (*p* < 0.05), with the same letters represent no significance difference (*p* > 0.05).

To determine whether the inhibition of adipocyte differentiation by catechin is mediated by AMPK-SIRT1 pathway, TNF-α induced adipocytes were treated with various concentrations of catechin (10–100 μg/mL). The protein levels of phosphorylated AMPK (Thr172) and phosphorylated SIRT1 (Ser 27) in TNF-α induced adipocytes were determined by Western blot (Fig 5). TNF-α (10 ng/mL) caused a significant increase of AMPK and SIRT1 in 3T3-L1 adipocytes. TNF-α induced adipocytes at the presence of 10–100 μg/mL catechin exhibited a dose-dependent inhibition on AMPK and SIRT1. However, the phosphorylation levels of AMPK and SIRT1 decreased 91% and 46%, respectively was observed in 3T3-L1 after incubated with TNF-α for 48 h. Adipocytes treated with catechin showed a does-dependent increase of TNF-α induced phosphorylation level for AMPK and SIRT1. These results suggest that catechin can modulate pro-inflammatory and anti-inflammatory cytokines by activating the AMPK/SIRT1 pathway.

### NF-κB-FOXO3a pathway is associated with catechin in 3T3-L1 adipocytes

**Fig 6:**
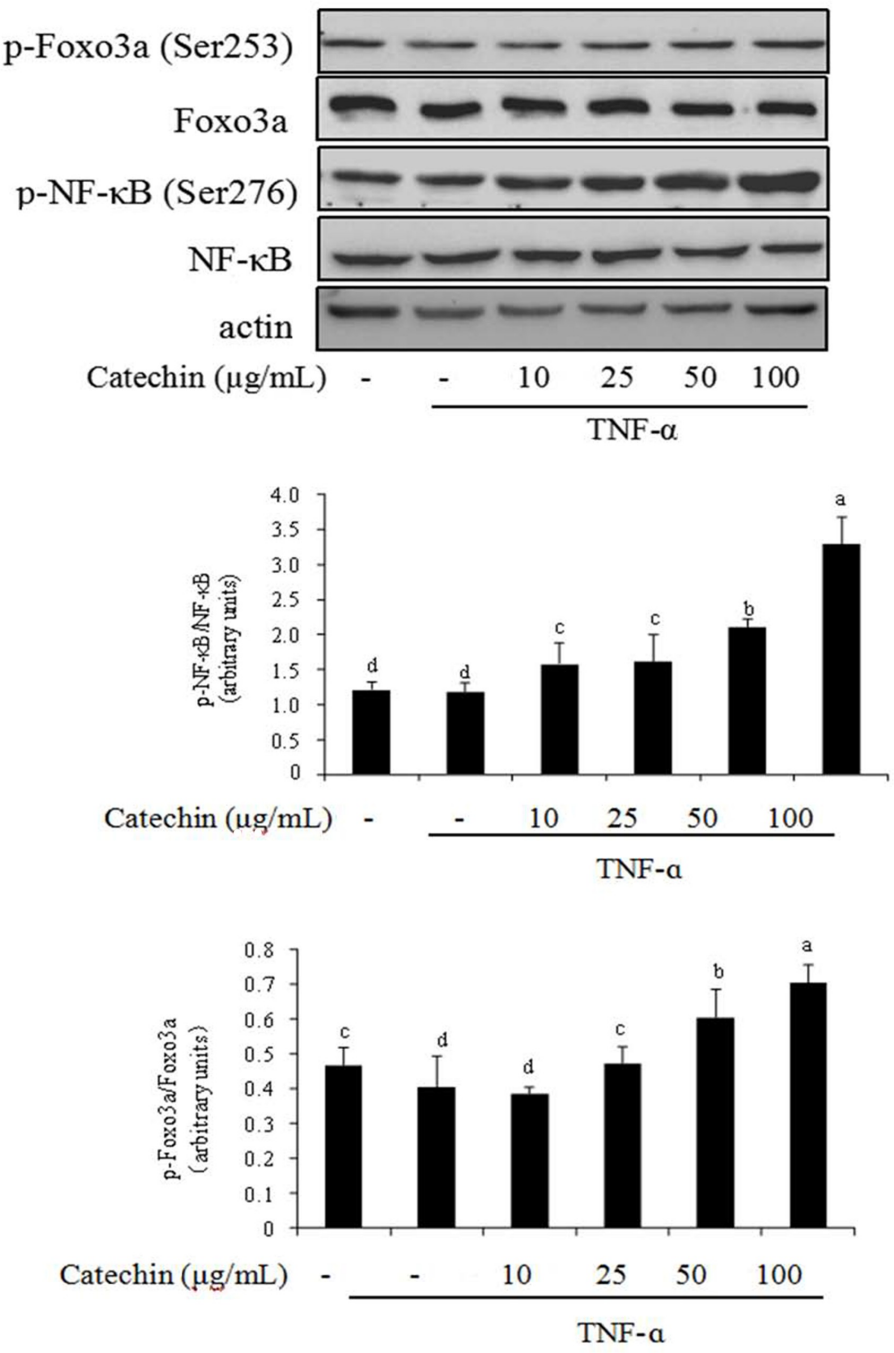
The effects of catechin on the activation of phosphorylation of NF-κBp65 and Foxo3a inhibited by TNF-α (10 ng/mL) in 3T3-L1 adipocytes. Data bars with the unlike letters represent the significance differences (*p* < 0.05), with the same letters represent no significance difference (*p* > 0.05).

The NF-κB signaling, at the core of chronic inflammation, is involved in both the innate and adaptive immune systems [26]. FOXO3a is also involved in the regulation of inflammation. Thus, we investigated the effect of catechin on the phosphorylation level of NF-κB and FOXO3a in TNF-α induced adipocytes (Fig 6). TNF-α caused an increase on the expression levels of p65 subunit of NF-κB. By contrast, the phosphorylation level of p65 decreased in 3T3-L1 adipocytes. When catechin was added to the media, the expression levels of p65 diminished. However, catechin (10–100 μg/mL) stimulated the increase on the phosphorylation level of p65 in a does-dependent manner. FOXO3a is an evolutionary conserved transcription factor which is involved in the regulation of inflammation [27]. The effect of catechin on the expression levels of FOXO3a and phosphorylated FOXO3a (Ser 253) was similar to p65 subunit of NF-κB in TNF-α induced adipocytes.

## Discussion

Inflammation, especially chronic inflammation, is an important defense response to resist pathogen invasion and repair tissue damage. Chronic inflammation and cellular senescence are inseparable in the process of accelerated or premature aging [28]. This is due to the associated production of pro-inflammatory cytokines (such as IL-1, IL-6, IL-12), anti-inflammatory cytokines (such as IL-4, IL-10) and inflammatory enzymes (such as iNOS, COX-2). These cytokines and enzymes drive the regeneration and activation of immune cells that achieve a balance between inflammatory and metabolic functions [29]. Natural products can provide abundant resources for anti-inflammatory compounds. Some dietary polyphenols, such as resveratrol, catechin, and quercetin, have been shown to be effective in preventing or alleviating inflammatory [22, 30]. The current work demonstrated that catechin had a capacity to protect adipocytes from TNF-α deleterious, with the increasing of the cell adipogenesis and viability. Catechin in the range of 10–100 μg/mL inhibited TNF-α induced the expression of genes involved in pro-inflammatory cytokines (IL-1, IL-6, IL-12) and inflammatory enzymes (iNOS, COX-2), and promoted TNF-α induced the expression of genes involved in anti-inflammatory cytokines (IL-4, IL-10). IL-4 and IL-10 are the protective cytokines against TNF-α induced inflammation. Vazquezprieto *et al*. [25] showed that TNF-α dependent transcription of pro-inflammatory genes (IL-6, resistin) was inhibited by (-)-epicatechin, which was consistent with our results. Similar result was also detected in the report from Chia *et al*. [6].

To get deeper insights in the mechanism of catechin on TNF-α induced adipocytes, we further researched cellular signal transduction in 3T3-L1 adipocytes and payed much attention to the AMPK-SIRT1 pathway. It has been reported that AMPK and SIRT1 have synergistic function in transcriptional factors and signal transduction proteins of inflammation response [31]. The activation of SIRT1 and AMPK significantly reduces the production of inflammatory factors and further inhibites the inflammatory response [32]. The activation of AMPK, SIRT1, FOXO3a and NF-κB signaling by TNF-α has been reported to cause inflammation and insulin resistance in several cell lines [33]. It has been showed that dietary polyphenols have an obvious anti-inflammatory effect through the pathway of AMPK/SIRT1-induced p65 deacetylation of NF-κB [16]. This study was to research that two energy target AMPK and SIRT1 serve as negative regulators of TNF-α induced inflammatory in 3T3-L1 adipocytes. In this experiment, TNF-α promoted the expression of AMPK, SIRT1, FOXO3a and NF-κB, but inhibited the expression of the phosphorylated AMPK (p-AMPK), phosphorylated SIRT1 (p-SIRT1), phosphorylated FOXO3a (p-FOXO3a) and phosphorylated NF-κB (p-NF-κB). In contrast, supplementation of catechin markedly increased the level of p-AMPK, p-SIRT1, p-FOXO3a and p-NF-κB, and gradually closed to the control group. Similar effects were observed for the extracts from grape [6], black tea [22], brown alga [34]. Consistent with our findings, a few studies have also demonstrated that synergistic activation of AMPK and SIRT1 can promote the expression of anti-inflammatory cytokines, and prevent the expression of pro-inflammatory cytokines via NF-κB signaling pathway in LPS-induced cells [35, 36].

SIRT1, as a downstream signal, mediates the anti-inflammatory effect of AMPK, which provides a mechanism of AMPK and SIRT1 in the inflammatory response [4]. The anti-inflammatory effect of AMPK is dependent on SIRT1, and changes in AMPK activity often affect the content and activity of SIRT1 [20]. Our results indicated that AMPK and SIRT1 also have effect on insulin sensitivity through their ability to antagonize TNF-α induced inflammation. Catechine supplementation remarkably reduced the phosphorylation level of AMPK, SIRT1, FOXO3a and NF-κB irrespective to the presence or absence of insulin. It seems that AMPK and SIRT1 synergistically regulate metabolic or inflammatory pathways. AMPK and SIRT1 may activate and feedback each other based on different cellular or physiological requirements. Activation of AMPK increases the expression of SIRT1 in 3T3-L1 adipocytes. Accordingly, activation of the AMPK-SIRT1-FoxO3a pathway by catechine may exert beneficial effects on inflammation treatment. Our study demonstrates the anti-inflammation effect of catechine in mature adipocytes through activation of the AMPK pathway, which consequently modulates gene expression of NF-κB through up-regulation of SIRT1 and FOXO3a.

In summary, these data demonstrate that catechin attenuates TNF-α stimulated inflammation by suppressing the activation of NF-κB and FOXO3a via AMPK/SIRT1 pathway, that lead to down-regulation of the gene expression of pro-inflammatory cytokines and inflammatory enzymes, and lead to up-regulating of the gene expression of anti-inflammatory cytokines. Regulation of AMPK/SIRT1 activity by catechin is a promising therapeutic strategy against many chronic inflammation and related diseases.

Adipose tissue/cells, as the initial site of systemic inflammation, play an important role in regulating energy metabolism in the body. Therefore, taking 3T3-L1 adipocytes as the model to study the mechanism of chronic inflammatory caused by metabolic disorders such as obesity and diabetes, which can be targeted for the prevention and treatment of metabolic inflammation, and also has important theoretical significance for revealing the anti-inflammatory mechanism of catechin.

## Abbreviations

**TNF-**α: tumor necrosis factor α
NF-κB: nuclear factor κB
AMPK: AMP-activated protein kinase
FOXO: forkhead box O
SIRT1: sirtuin1
p-AMPK: phosphorylated AMPK
p-SIRT1: phosphorylated SIRT1
p-FOXO3a: phosphorylated FOXO3a
p-NF-κB: phosphorylated NF-κB
COX-2: cyclooxygenase-2
IL: interleukin
iNOS: inducible nitric oxide synthase
FBS: Fetal bovine serum
DMEM: Dulbecco's modified Eagle's medium
IBMX: 3-isobutyl-1-methylxanthine
DEX: dexamethasone
CCK-8: Cell Counting Kit-8
GAPDH: glyceraldehyde-3-phosphate dehydrogenase
LPS: lipopolysaccharide
SDS-PAGE: sodium dodecyl sulfate-polyacrylamide gel electrophoresis
